# Health-related quality of life and functional recovery one year after emergency abdominal surgery in patients over 75 years: a prospective observational study

**DOI:** 10.3389/fsurg.2026.1852532

**Published:** 2026-07-10

**Authors:** Elin Kismul Aakre, Atle Ulvik, Gabriele Leonie Schwarz, Bjørn Steinar Olden Nedrebø, Elisabeth Skaar, Katinka Alme, Ib Jammer

**Affiliations:** 1 Department of Anaesthesia and Intensive Care, Haukeland University Hospital, Bergen, Norway; 2 Department of Clinical Medicine, University of Bergen, Bergen, Norway; 3 Department of Gastrointestinal Surgery, Haukeland University Hospital, Bergen, Norway; 4 Clinic of Heart Disease, Haukeland University Hospital, Bergen, Norway; 5 Department of Clinical Science, University of Bergen, Bergen, Norway; 6 Clinic of Internal Medicine, Haukeland University Hospital, Bergen, Norway

**Keywords:** aged, geriatric surgery, health-related quality of life (HRQL), long-term outcomes, patient reported outcome measures

## Abstract

**Introduction:**

Emergency abdominal surgery is common in older patients, yet data on long-term patient centered outcomes are scarce, limiting informed decision making for this vulnerable population. The aim of this study was to investigate functional outcomes in older patients one year after emergency abdominal surgery, including residence status, perceived health, willingness to repeat the procedure, and health-related quality of life.

**Methods:**

Single-center cohort study of patients aged ≥75 years undergoing emergency abdominal surgery at a Norwegian university hospital. Preoperatively, patients were assessed for frailty (Clinical Frailty Scale), comorbidity (Charlson Comorbidity Index) and preoperative residence status. Survivors were followed up after one year by telephone. Health-related quality of life was assessed by the EuroQol Five Dimensions Three Levels (EQ-5D-3L) questionnaire.

**Results:**

Of 154 patients undergoing emergency abdominal surgery, 135 (88%) were discharged alive from hospital. At follow-up, 106/154 (69%) were alive. Of those, 74 (70%) completed the questionnaire. The follow-up cohort was younger [median age 79 [IQR 77–84] vs. 83 [IQR 79–88]; *p* < 0.001], had lower Charlson Comorbidity Index [5 [IQR 4–6] vs. 6 [IQR 5–8]; *p* < 0.001] and lower Clinical Frailty Scale score [3 [IQR 2–4] vs. 4 [IQR 3–6]; *p* < 0.001] compared to the non-assessable cohort (*n* = 80). In the follow-up cohort, 71/74 (96%) had returned home. For the EQ-5D dimensions, 32/74 (43%) participants reported problems with mobility, 9/74 (12%) with self-care, and 24/74 (36%) with usual activities. Some or extreme pain or discomfort were reported by 34/74 (46%) and anxiety or depression by 25/74 (34%) participants. Respondents reported a mean EQ-5D visual analogue scale score of 68 ± 20 (SD). 53/73 (73%) reported improved or unchanged health status, and 57/74 (77%) were willing to undergo emergency abdominal surgery again.

**Conclusion:**

One year after emergency abdominal surgery in older patients, half of the patients were alive and able to report their recovery status. Functional recovery and health-related quality of life were comparable to Norwegian reference populations, and three of four would choose emergency surgery again. The findings are subject to selection and response biases, as the frailest patients were unable to participate or did not survive to follow-up assessment.

## Introduction

Evidence on health-related quality of life in older patients who have emergency abdominal surgery is limited, even though more than half of those undergoing the procedure are over 65 years of age (1). Most studies report traditional outcomes such as mortality and length of stay, while patient-centred outcomes - including patient's perceptions of their health, functioning, and quality of life - are rarely reported. Existing studies predominately include younger patients, leaving outcomes beyond hospital discharge poorly characterized in the oldest age groups (2–9). To support informed decision making and patient-centred care, clinicians need data on long-term functional outcomes and quality of life after abdominal surgery in older patients. We aim to address this knowledge gap by investigating residence status, treatment acceptability and health-related quality of life, one year after emergency abdominal surgery in patients aged ≥75 years.

## Material and methods

This is a sub-study of the Protocol for Patients Above 75 Years Undergoing Emergency Laparotomy (ProPEL) study (10), approved by the Western Norway Regional Ethics Committee (REK-Vest ID 2019-7110) and registered at https://www.clinicaltrials.gov (NCT04293653). The results are reported according to the CONSORT checklist for patient reported outcomes (11). Informed consent was obtained from participants as soon as feasible after emergency abdominal surgery. For patients with cognitive impairment, proxy consent was sought from the patient's next-of-kin.

### Setting and participants

At Haukeland University Hospital, Bergen, Norway, consecutive patients aged 75 years or older who underwent emergency abdominal surgery between January 2020 and April 2021 were included. The hospital provides elective and emergency gastrointestinal surgery for a referral population of approximately 500 000 inhabitants. Emergency abdominal surgery was defined as any gastrointestinal pathology requiring surgical intervention within 72 h. All patients were managed according to a standardised care pathway that included preoperative frailty assessment, shared decision-making with palliation offered as an alternative for severely frail patients, hemodynamic optimization, and protocolized intraoperative care. Details of the pathway and its impact on short-term outcomes are reported elsewhere (10). Patients undergoing laparoscopic appendectomy, surgery for known inoperable malignancy, simple hernia repair or vascular surgery were excluded. One year after surgery, follow-up was undertaken by a single investigator (EKAA) using a standardised telephone interview. The interview included questions regarding patients' residence status, the EuroQol Five Dimensions Three Levels (EQ-5D-3L) questionnaire and two self-developed questions on perceived health status and willingess to undergo surgery again.

Potential participants were approached up to five times on different days and hours to maximise response rates. Patients who were unable to communicate by telephone due to hearing impairment, aphasia or cognitive impairment were excluded from follow-up at the investigator's discretion.

### Data collection

Data were collected prospectively, except for the Charlson Comorbidity Index (CCI) which was scored retrospectively based on electronic patient records. Frailty was assessed using the Clinical Frailty Scale (CFS) (12). According to the CFS, patients are stratified as fit (CFS score 1–3), vulnerable (CFS score 4) or frail (CFS score 5–9).

The EQ-5D-3L is a generic questionnaire developed and validated to investigate self-reported health-related quality of life (13). The tool consists of two parts, the descriptive EQ-5D-3L system and the EQ-5D visual analogue scale (EQ-5D-VAS). The descriptive system (EQ-5D-3L) explores five dimensions of health: mobility, self-care, usual activities, pain/discomfort, and anxiety/depression - each rated on a three-level Likert scale: no problems, some problems, and extreme problems. The EQ-5D-VAS measures self-rated general health status, asking respondents to rate “your own health state today” on a scale from 0 to 100. Endpoints are labelled “Best imaginable health state” (100 points) and “Worst imaginable health state” (0 points).

To assess patient's perceived recovery and willingness to undergo the surgical procedure again, two self-developed closed questions were asked:

1. “Compared to your health before the operation, would you say your health today is about the same, better, or worse?” Responses were “unchanged”, “better” or “worse”.
2. “If you needed the same operation and treatment again, how likely would you consent?”

Responses were “likely”, “unsure” and “unlikely”.

### Statistical analysis

Categorical variables are reported as counts and percentages. Continuous data are reported as medians with interquartile range, IQR [25th–75th percentiles] or means and standard deviations (SD). Comparisons were made using the chi-square test for categorical variables and the Mann–Whitney test for continuous variables. The statistical analyses were conducted in SPSS version 24 software (Chicago, IL, USA).

## Results

**Figure 1:**
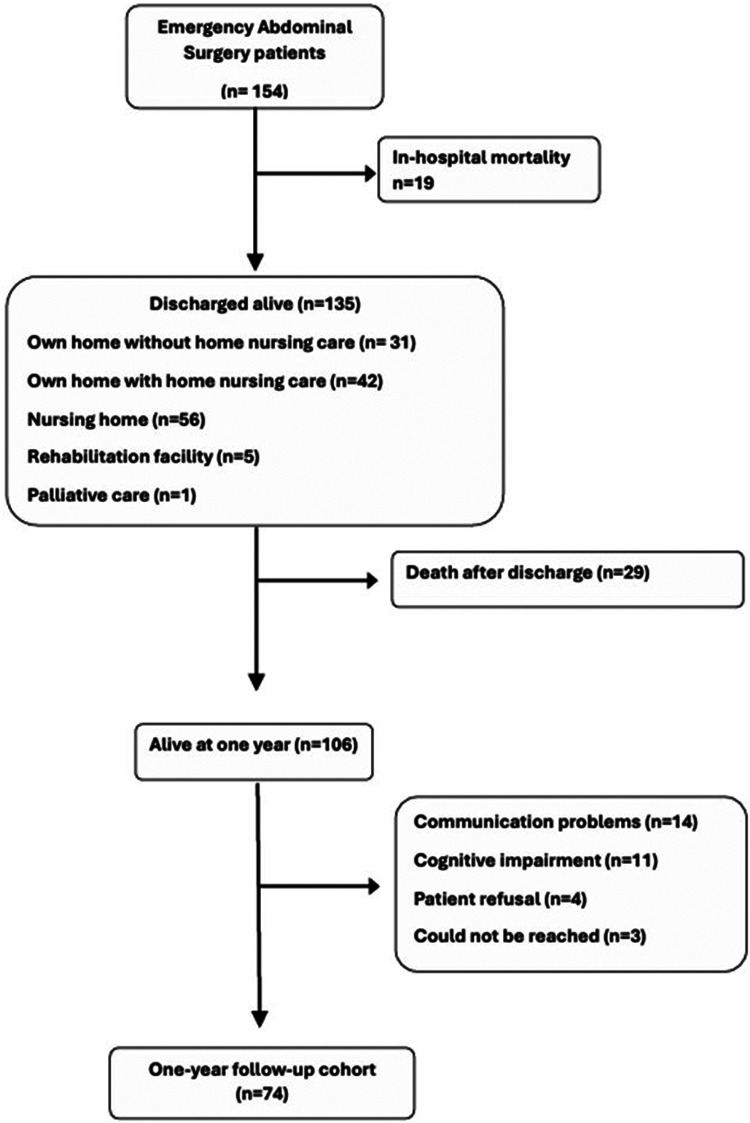
Patient flow diagram. One year after surgery, 106 patients were alive, and of these, 74 (70%) were able to report their recovery status. This cohort represents the healthiest survivors and comprises half of the population undergoing emergency abdominal surgery.

Of 154 patients undergoing emergency abdominal surgery, 135 (88%) were discharged from hospital alive and 106 (69%) were alive at one year (Figure 1). Of those alive at one year after surgery, 74 patients completed a telephone-based interview (response rate 74/106; 70%). This follow-up cohort represented 48% of the original cohort of older patients undergoing emergency abdominal surgery.

Death, communication barriers and cognitive impairment where the most common reasons patients could not report their health-related quality of life (Figure 1). Four patients refused to participate in the questionnaire and three could not be reached. The follow-up cohort was significantly younger, with fewer comorbidities (in particular, cognitive impairment and dementia), and lower levels of frailty, compared to non-assessable patients (*n* = 80, Table 1).

**Table 1:** Baseline preoperative characteristics of the follow-up cohort (*n* = 74) and the non-assessable cohort (*n* = 80).

Variable		Follow-up cohort, *n* = 74	Non-assessable cohort, *n* = 80	*p*-value
Age (years), median (IQR), range		79 (77–84), 75–96	83 (79–88), 75–97	<0.001^a^
Female, *n* (%)		43 (58)	40 (50)	0.31^b^

**Comorbidities, *n* (%)**				
**Ischaemic heart disease, heart failure, arrythmia, pacemaker, peripheral vascular disease**		36 (49)	44 (55)	0.431^b^
Glomerular filtration rate <60 mL/min		33 (45)	43 (54)	0.256^b^
Chronic obstructive pulmonary disease/asthma		19 (26)	21 (26)	0.935^b^
Diabetes mellitus		7 (10)	12 (15)	0.296^b^
Cancer, other than gastrointestinal		7 (10)	13 (16)	0.21^b^
Dementia/ cognitive impairment		3 (4)	19 (24)	<0.001^b^
Ischemic stroke (with sequel)		1 (1)	9 (11)	0.013^b^

Daily medication, median (IQR)		5 (3–7)	5 (4–8)	0.102^a^

**Preoperative residence**				<0.001^b^
Own home without home nursing care		65 (88)	46 (58)	
Own home with home nursing care		5 (7)	22 (28)	
Assisted living facility		4 (5)	7 (9)	
Nursing home		0 (0)	5 (6)	

**ASA physical status**, median (IQR)		3 (2–3)	3 (3–4)	
**ASA Category**, *n* (%)				<0.001^b^
	ASA 1	0 (0)	1 (1)	
	ASA 2	22 (30)	5 (6)	
	ASA 3	44 (60)	48 (60)	
	ASA 4	8 (11)	26 (33)	
**Preoperative National Early Warning Score (NEWS),** median (IQR)		2.5 (1–5)	3 (1–6)	0.307^a^
**Charlson Comorbidity Index**, median (IQR)		5 (4–6)	6 (5–8)	<0.001^a^

**Clinical Frailty Scale**, median (IQR)		3 (2–4)	4 (3–6)	<0.001^a^
**Clinical Frailty Scale Category**, *n* (%)				<0.001^b^
	CFS 1	9 (12)	2 (3)	
	CFS 2	15 (20)	8 (10)	
	CFS 3	26 (35)	12 (15)	
	CFS 4	17 (23)	24 (30)	
	CFS 5	4 (5)	12 (15)	
	CFS 6	3 (4)	18 (23)	
	CFS 7	0 (0)	4 (5)	

a Mann–Whitney test.

b Chi-square test.

At the time of surgery, the follow-up cohort had a median age of 79 years (range 75–96) and 58% were female. Serious comorbidity was frequent while frailty was uncommon with 7/74 (9%) having a CFS score ≥ 5 (Table 1). Before surgery, 70/74 (95%) of the patients in the follow-up cohort lived in their own home, of whom 5/74 (7%) received home nursing care. The most common procedures were colonic or small bowel resections (30% and 26%, respectively; Table 2). Median length of stay was nine [IQR 7–14] days and 53/74 (72%) were discharged to their own home with or without home nursing care. 19/74 (26%) were discharged to a short-term stay in a nursing home. At one-year follow-up, 71/74 patients (96%) had returned to live in their own homes and only 3/74 (4%) lived in assisted living facility (Table 3). One in four of those living at home received home nursing services.

**Table 2:** Surgical procedures, length of stay, and discharge destination of the follow-up cohort (*n* = 74) and the non-assessable cohort (*n* = 80).

Variable	One-year follow-up cohort, *n* = 74	Non-assessable cohort, *n* = 80	*p*-value
Indication for surgery			0.684^a^
Obstruction	46 (62)	46 (58)	
Sepsis	12 (16)	8 (10)	
Ischemia	2 (3)	3 (4)	
Bleeding	1 (1)	2 (3)	
Perforation	12 (16)	20 (25)	
Other (dehiscence, second-look procedure)	1 (1)	1 (1)	
Category of surgery			0.891^a^
Laparotomy completed	59 (80)	63 (79)	
Laparoscopy completed	5 (7)	7 (9)	
Laparoscopy intended, converted to open	10 (14)	10 (13)	
Reoperation (following elective procedure)	9 (12)	7 (9)	0.488^a^
Resection of organ			
Colon or rectum	22 (30)	17 (21)	
Small bowel	19 (26)	17 (21)	
Gallbladder	3 (4)	3 (4)	
Stomach	1 (1)	3 (4)	
Stoma formation	9 (12)	15 (19)	
Other procedure (laparotomy/scopy)	9 (12)	22 (28)	
Adhesiolysis	11 (15)	11 (14)	
Length of stay (days), median (IQR)	9 (7–14)	12 (7–17)	0.085^b^
Discharge destination			<0.001^a^
Own home	25 (34)	6 (8)	
Own home with assistance from health care personnel	28 (38)	14 (18)	
Nursing home	19 (26)	37 (46)	
Rehabilitation	2 (3)	3 (4)	
Palliative care	0	1 (1)	
In-hospital mortality	0	19 (24)	

Values are *n* (%) unless otherwise indicated.

a Chi-square test.

b Mann–Whitney test.

**Table 3:** Recovery status at one-year follow up (*n* = 74).

Variable			Response	
**Residence at one-year follow-up**				**Number (%)**
Own home without home nursing care				53 (72)
Own home with home nursing care				18 (24)
Assisted living facility				3 (4)
**EuroQol Five Dimensions (EQ-5D)**	**Missing**	**No problems**	**Some problems**	**Extreme problems**
Mobility	0 (0)	42 (57)	32 (43)	0 (0)
Self-Care	0 (0)	65 (88)	9 (12)	0 (0)
Usual activities	0 (0)	47 (64)	24 (32)	3 (4)
Pain/discomfort	0 (0)	40 (54)	17 (23)	17 (23)
Anxiety/depression	0 (0)	49 (66)	17 (23)	8 (11)
**EQ-5D Visual analogue scale** score mean, +/- (SD)	3 (0)			68 +/-(20)
**Self-developed questions**	**Missing**	**Unchanged**	**Better**	**Worse**
Self-rated health one year following emergency abdominal surgery	1 (0)	30 (41)	23 (32)	20 (27)
		**Yes**	**Unsure**	**No**
Willingness to undergo the same procedure again	0 (0)	57 (77)	13 (18)	4 (5)

Values are n (%) unless otherwise indicated.

**Figure 2:**
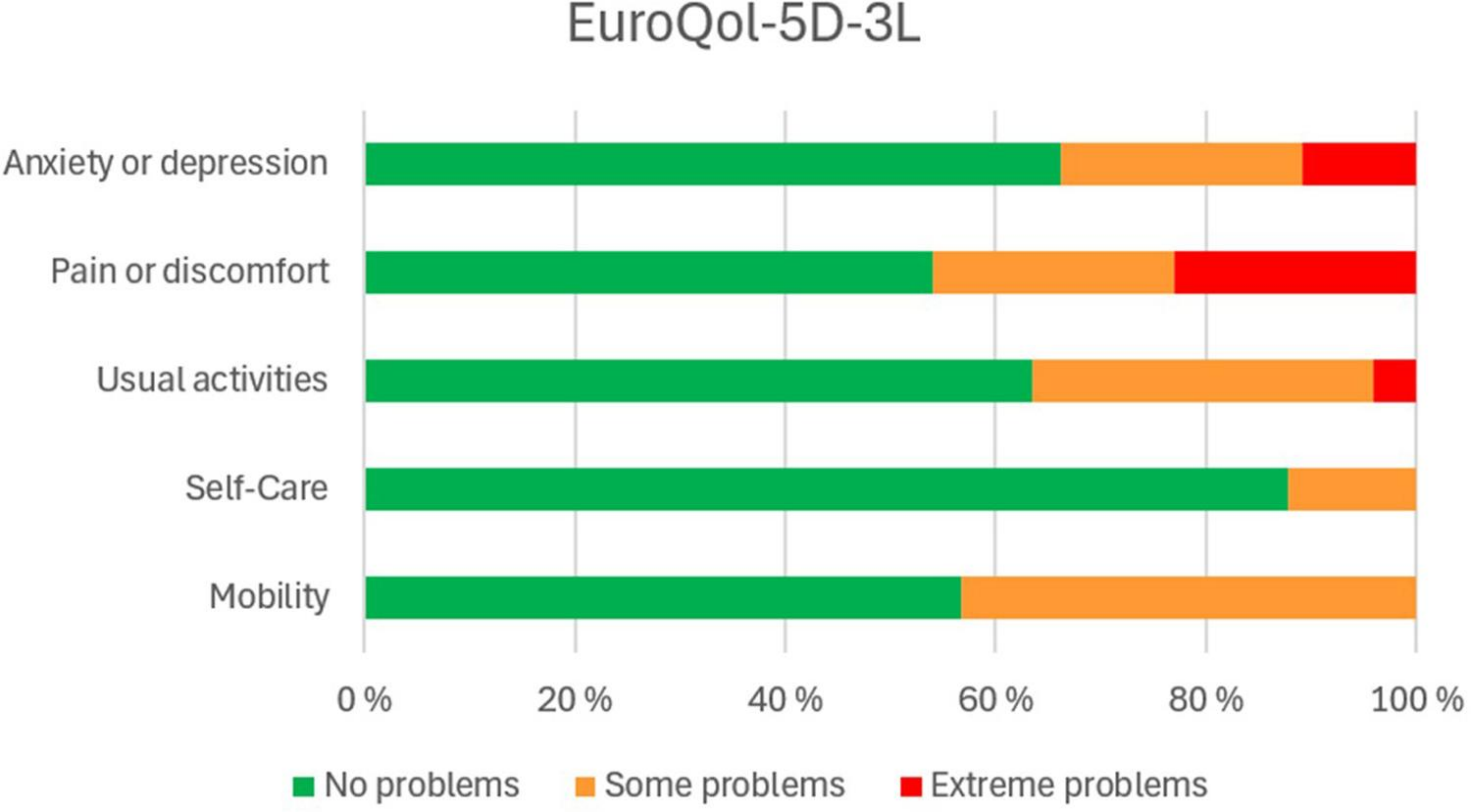
Self-reported health-related quality of life in the five dimensions and three levels of the EuroQol five dimensions three levels (EQ-5D-3L) questionnaire.

Follow-up data on EQ-5D-3L are presented in Figure 2 and Table 3. Problems were reported with mobilising (43%), self-care (12%), and performing usual activities (36%). Extreme pain or discomfort was relatively common (23%), and severe anxiety or depression was reported by 11%. The general health status, assessed by the EQ-5D-VAS, was mean (SD) 68 (20).

Overall, 53/73 (73%) rated their health as improved or unchanged compared with before surgery, and 57/74 (77%) stated that they would likely consent to the surgical procedure again if needed. Thirteen patients (18%) were unsure, and four (5%) would refrain from emergency abdominal surgery in the future (Table 3).

## Discussion

One year after emergency abdominal surgery in older patients, fewer than half of the patients were alive and able to report measures of recovery. Of this cohort, almost all patients had returned to live at home. The majority reported no problems with walking, self-care or performing their usual activities. Although some patients reported severe pain/discomfort, and/or severe anxiety/depression, 73% described their health status as improved or unchanged, and 77% were willing to undergo emergency abdominal surgery in the future. Importantly, the frailest patients did not survive to one-year follow-up and therefore, their health-related quality of life could not be assessed.

The EQ-5D-3L has been employed to investigate health status after emergency abdominal surgery (4, 7, 14). For Norway, a population norm for the EQ-5D-3L has been published (15), in which respondents are aged ≥ 71 years, hence slightly younger than our cohort. Additionally, the EQ-5D-3L was used in a Norwegian population sample (*n* = 179) with a mean age of 86.7 years (16). Our patients (aged 75–96 years; median age 79) fall between these two cohorts and showed comparable results. Unsurprisingly, the responses related to the domains of mobility, self-care and usual activities of our cohort lie between those of the younger (15) and older (16) Norwegian reference populations. For mobility, 43% of our cohort reported some problems, compared to 35% (15) and 56% (16), respectively. The same pattern was seen with self-care [some problems: 12%, compared with 6% (15) and 25% (16)] and for the ability to perform usual activities [some/extreme problems: 36%, compared with 28% (15) and 50% (16)].

Pain or discomfort was reported in 46% of our patients, which is lower than the general Norwegian population [61% (15) and 65% (16)]. Chronic pain is common after abdominal surgery. Following surgery for small bowel obstruction (17), chronic postsurgical pain was reported by 21% of the patients and affected daily functioning in 19%. These findings were confirmed in a study involving 440 patients undergoing emergency laparotomy, where 19% complained of chronic pain and 45% experienced functional impairment to some extent (18). These differences might be due to case mix or response bias, i.e., the survivors with the best results being more likely to respond to the survey.

Anxiety or depression was reported by 34%, which is slightly higher than the 19% (15) to 30% (16) reported among older Norwegians, but in keeping with having experienced critical illness. Following life-threatening illness, emotional distress is common, and “extreme anxiety or depression” was reported by up to 47% in a study of older ICU survivors (19) (median age 83).

Our cohort reported their general health status, measured using the EQ-5D-VAS, with a mean score of 68 (SD 20), nearly identical to Norwegian population norms for older adults [68 (15) and 67.7 (16)]. This contrasts with a British study (20) reporting lower physical functioning in emergency laparotomy survivors compared with the general population. These differences may reflect methodological variation (different assessment tools), healthcare system differences, or selection bias in both studies.

Living at home and being functionally independent are recognised as some of the most important factors for health and well-being in advanced age (21–23). Patients' perspective on what constitutes a good recovery is not necessarily captured by traditional parameters such as mortality or postoperative complications, and older patients may place greater value on functional or cognitive independence (23). Studies confirm the importance of resuming pre-surgical activities, symptom relief, being able to enjoy life, and regaining independence as key elements for subjective recovery (21, 24).

Our cohort reported notable levels of pain, discomfort and anxiety, indicating a substantial impact of emergency abdominal surgery on physical and mental health (3). Nevertheless, 77% of the patients were willing to undergo the same procedure again if indicated, suggesting that the treatment burden seemed acceptable in hindsight. This finding aligns with another Nordic study investigating patients' willingness to undergo an emergency surgical procedure again, in which 73% considered it likely they would do so (25). Comparable results have been reported in a cohort of octogenarians surviving emergency abdominal surgery (26). Similarly, a Dutch study of older ICU survivors after abdominal sepsis found that, even though fewer than half had regained their baseline function, 94% would undergo ICU treatment again (27), consistent with findings reported elsewhere (28).

We assessed patient-reported outcomes one year after surgery; however, we lack information on patients' health transitions during that year. Perceived health status varies during the postoperative course and may return to baseline over time (7, 8). One study of a comparable Danish cohort demonstrated that following emergency abdominal surgery, functional status shows an immediate decline before returning to baseline within 180 days postsurgery (5). Our study confirms that among survivors with good functional outcomes, significant improvement occurred during the first year after emergency abdominal surgery, with almost all of them eventually returning to their homes.

How survivors with poor functional status reflect on the benefit or harm of emergency abdominal surgery is difficult to evaluate. In our study, survivors who were unable to participate in the follow-up questionnaire due to sensory, expressive or cognitive impairment comprised approximately 15% of all patients who underwent emergency abdominal surgery. Additionally, health-related quality of life among those who survived surgery and hospitalisation but died within one year remains unknown.

When faced with life-threatening illness, many older patients and their next-of-kin perceive “choosing life”, including the burdens of aggressive treatment, as the only option (29–31). At the same time, older patients may wish both to survive with what they regard as a good outcome and to achieve a dignified end of life. Moreover, evidence suggests that some older patients in Western societies may consider survival with poor physical and/or cognitive function as an outcome worse than death (32–34).

To summarise our findings, long-term functional recovery (including mobility, self-care and resumption of usual activities) and quality of life among survivors were comparable with those of the background population. Most survivors were willing to undergo the procedure again, suggesting good overall acceptability of the intervention. However, these findings are limited to long-term survivors, who represent the healthiest and most physically fit patients, suggesting that long-term outcomes in this group are favorable.

This study has several important limitations. First, substantial selection and response biases limit generalisation to real-world geriatric populations. One-year mortality was high (31%), and frail patients did not survive to follow-up. Furthermore, patients with cognitive or communicative impairments (approximately 15% of the original cohort) were excluded, potentially representing those with poorer outcomes. Our findings therefore reflect the experience of healthier survivors, who represent only 48% of the original cohort, and cannot be extrapolated to the entire population undergoing emergency abdominal surgery.

Second, the lack of baseline EQ-5D data limits our ability to assess true change in quality of life. Third, the single assessment at one year does not capture recovery trajectories or the burden experienced by patients who died between discharge and one year. From this study, we learnt that future research should include longitudinal follow-up after emergency abdominal surgery, with frequent collection of patient-reported functional outcomes, to better understand the trajectories of very old patients, particularly those with less favorable outcomes. Furthermore, the study was not designed to identify risk factors associated with poor functional recovery or reduced health-related quality of life. Fourth, our two self-developed questions assessing health change and treatment acceptability were not formally validated. Additionally, asking participants to compare their current health status with how they remember their health before undergoing emergency abdominal surgery one year earlier is relevant from a patient perspective, but may have introduced recall bias as we did not assess health-related quality of life at inclusion. Fifth, the single-centre design and Norwegian healthcare context may limit generalisability to other settings.

Despite small numbers, our study provides detailed descriptions of both the follow-up cohort and patients who were non-assessable, with few missing data, and addresses patient reported outcomes relevant to a large population which is only scarcely studied so far. Our findings may create the base for further quality improvement efforts and intervention studies. Larger studies are needed to address these crucial aspects of emergency surgical treatment concerning a vulnerable, large and rapidly growing population.

## Conclusion

In our study of older Norwegian patients, the health-related quality of life of survivors one year after emergency abdominal surgery was comparable to that of the general older population, and most survivors expressed willingness to undergo the procedure again. These findings are derived from less than half of the cohort presenting for emergency surgery and are therefore subject to selection and response biases, as the frailest patients were unable to participate or did not survive to follow-up.

## Data Availability

The raw data supporting the conclusions of this article will be made available by the authors, without undue reservation.

## References

[1] NELA Project Team. Eighth Patient Report of the National Emergency Laparotomy Audit. Royal College of Anaesthetists (RCoA) London (2023)

[2] Holmes M Rugendyke A Ming YJ Howley P Gani J Pockney P. Getting back “home” after emergency laparotomy: how many never make it? ANZ J Surg. (2023) 93(10):2433–8. 10.1111/ans.18685 37675923

[3] McIlveen EC Edwards J Vella M McKinlay L Hancock C Quasim T. Patient-reported impact of emergency laparotomy on employment and health status 1 year after surgery. Langenbecks Arch Surg. (2023) 408(1):378. 10.1007/s00423-023-03104-y 37749405

[4] Kwong E Neuburger J Murray D Black N. Feasibility of collecting and assessing patient-reported outcomes for emergency admissions: laparotomy for gastrointestinal conditions. BMJ Open Gastroenterol. (2018) 5(1):e000238. 10.1136/bmjgast-2018-000238 30397506 PMC6203065

[5] ÍS L Kokotovic D Kvist M Hansen JB Burcharth J. Long-term impact of emergency laparotomy on health-related quality of life. Eur J Trauma Emerg Surg. (2025) 51(1):40. 10.1007/s00068-024-02745-y 39853378 PMC11761775

[6] Kokotovic D ÍS L Hansen TL Knoblauch JB Balle CB Jensen L. Impact of a transition of care bundle on health-related quality of life after major emergency abdominal surgery: before-and-after study. BJS Open. (2025) 9(2):zraf020. 10.1093/bjsopen/zraf020 40099557 PMC11914972

[7] Gordeev VS Assefa E Pearse R Edwards M Mihaylova B. Health-related quality of life after emergency abdominal surgery. World J Emerg Surg. (2025) 20(1):73. 10.1186/s13017-025-00643-1 40898348 PMC12403461

[8] Khanderia E Aggarwal R Bouras G Patel V. Quality of life after emergency laparotomy: a systematic review. BMC Surg. (2024) 24(1):73. 10.1186/s12893-024-02337-y 38409008 PMC10898072

[9] El-Kefraoui C Do U Miller A Kouyoumdjian A Cui D Khorasani E. Impact of enhanced recovery pathways on patient-reported outcomes after abdominal surgery: a systematic review. Surg Endosc. (2023) 37(10):8043–56. 10.1007/s00464-023-10289-2 37474828

[10] Aakre EK Nedrebø BSO Ulvik A Ranhoff AH Flaatten H Hufthammer KO. Impact of a care pathway for older patients undergoing emergency abdominal surgery: a before-and-after study. Acta Anaesthesiol Scand. (2026) 70(2):e70182. 10.1111/aas.70182 41517929 PMC12789884

[11] Calvert M Blazeby J Altman DG Revicki DA Moher D Brundage MD. Reporting of patient-reported outcomes in randomized trials. JAMA. (2013) 309(8):814. 10.1001/jama.2013.879 23443445

[12] Rockwood K Song X MacKnight C Bergman H Hogan DB McDowell I, et al. A global clinical measure of fitness and frailty in elderly people. Can Med Assoc J. (2005) 173(5):489–95. 10.1503/cmaj.050051 16129869 PMC1188185

[13] Rabin R de Charro F. EQ-5D: a measure of health status from the EuroQol group. Ann Med. (2001) 33(5):337–43. 10.3109/07853890109002087 11491192

[14] Gazala S Tul Y Wagg A Widder SL Khadaroo RG. Quality of life and long-term outcomes of octo- and nonagenarians following acute care surgery: a cross sectional study. World J Emerg Surg. (2013) 8(1):23. 10.1186/1749-7922-8-23 23816269 PMC3734003

[15] Stavem K Augestad LA Kristiansen IS Rand K. General population norms for the EQ-5D-3 L in Norway: comparison of postal and web surveys. Health Qual Life Outcomes. (2018) 16(1):204. 10.1186/s12955-018-1029-1 30340499 PMC6194590

[16] Andersen FH Flaatten H Klepstad P Romild U Kvale R. Long-term survival and quality of life after intensive care for patients 80 years of age or older. Ann Intensive Care. (2015) 5(1):53. 10.1186/s13613-015-0053-0 26055187 PMC4456598

[17] Jeppesen M Tolstrup MB Gögenur I. Chronic pain, quality of life, and functional impairment after surgery due to small bowel obstruction. World J Surg. (2016) 40(9):2091–7. 10.1007/s00268-016-3616-9 27384171

[18] Tolstrup MB Thorup T Gögenur I. Chronic pain, quality of life and functional impairment after emergency laparotomy. World J Surg. (2019) 43(1):161–8. 10.1007/s00268-018-4778-4 30178128

[19] Ferrão C Quintaneiro C Camila C Aragão I Cardoso T. Evaluation of long-term outcomes of very old patients admitted to intensive care: survival, functional status, quality of life, and quality-adjusted life-years. J Crit Care. (2015) 30(5):1150.e7–11. 10.1016/j.jcrc.2015.05.005 26143283

[20] Alder L Mercer SJ Carter NC Toh SK Knight BC. Clinical frailty and its effect on the septuagenarian population after emergency laparotomy. Ann R Coll Surg Engl. (2021) 103(3):180–5. 10.1308/rcsann.2020.7028 33645274 PMC9158232

[21] Rajabiyazdi F Alam R Pal A Montanez J Law S Pecorelli N. Understanding the meaning of recovery to patients undergoing abdominal surgery. JAMA Surg. (2021) 156(8):758–65. 10.1001/jamasurg.2021.1557 33978692 PMC8117063

[22] Dragosits A Martinsen B Hemingway A Norlyk A. Coming home: older patients' And their relatives' Experiences of well-being in the transition from hospital to home after early discharge. Int J Qual Stud Health Well-being. (2024) 19(1):2300154. 10.1080/17482631.2023.2300154 38166522 PMC10769116

[23] Fried TR Bradley EH Towle VR Allore H. Understanding the treatment preferences of seriously ill patients. N Engl J Med. (2002) 346(14):1061–6. 10.1056/NEJMsa012528 11932474

[24] Law J Welch C Javanmard-Emamghissi H Clark M Bisset CN O'Neil P. Decision-making for older patients undergoing emergency laparotomy: defining patient and clinician values and priorities. Colorectal Dis. (2020) 22(11):1694–703. 10.1111/codi.15165 32464712

[25] Tengberg LT Foss NB Lauritsen ML Orbæk J Hulvej Rod M Tjørnhøj-Thomsen T. The impact of acute high-risk abdominal surgery on quality of life in elderly patients. Dan Med J. (2017) 64(6):A5371. PMID: 28566117

[26] Hale E Davis H Sert S Bowling K. WP5.9 - Emergency laparotomies in the elderly: a patient perspective. BJS. (2024) 111(Supplement_8):znae197.172. 10.1093/bjs/znae197.172

[27] Cuijpers ACM Coolsen MME Schnabel RM Lubbers T van der Horst ICC van Santen S. Self-perceived recovery and quality of life in elderly patients surviving ICU-admission for abdominal sepsis. J Intensive Care Med. (2022) 37(7):970–8. 10.1177/08850666211052460 34756128 PMC9136475

[28] Schrøder MA Poulsen JB Perner A. Acceptable long-term outcome in elderly intensive care unit patients. Dan Med Bull. (2011) 58(7):A4297. PMID: 21722543

[29] Lound A Bruton J Jones K Shah N Williams B Gross J. I'd rather wait and see what's Around the corner": a multi-perspective qualitative study of treatment escalation planning in frailty. PLoS One. (2023) 18(9):e0291984. 10.1371/journal.pone.0291984 37733669 PMC10513333

[30] Schwarz GL Sjobo BA Skaar E Miljeteig I Burns KEA Kvåle R. I am old, and I will rather abandon life with my eyes open': a qualitative study of norwegians aged 80 and older explaining their intensive care unit admission preferences. Age Ageing. (2025) 54(10):afaf297. 10.1093/ageing/afaf297 41091696 PMC12526999

[31] Warner BE Harry A Wells M Brett SJ Antcliffe DB. Escalation to intensive care for the older patient. An exploratory qualitative study of patients aged 65 years and older and their next of kin during the COVID-19 pandemic: the ESCALATE study. Age Ageing. (2023) 52(4):afad035. 10.1093/ageing/afad035 37083851 PMC10120351

[32] Aronson L. Beyond code Status. N Engl J Med. (2024) 390:1451–3. 10.1056/NEJMp2314068 38647052

[33] Teater B Chonody JM. How do older adults define successful aging? A scoping review. Int J Aging Hum Dev. (2020) 91(4):599–625. 10.1177/0091415019871207 31456410

[34] Lee RY Brumback LC Sathitratanacheewin S Lober WB Modes ME Lynch YT. Association of physician orders for life-sustaining treatment with ICU admission among patients hospitalized near the end of life. JAMA. (2020) 323(10):950–60. 10.1001/jama.2019.22523 32062674 PMC7042829

